# Molecular Confirmation of Bacillus Calmette Guerin Vaccine Related Adverse Events among Saudi Arabian Children

**DOI:** 10.1371/journal.pone.0113472

**Published:** 2014-11-19

**Authors:** Sahal Al-Hajoj, Ziad Memish, Naila Abuljadayel, Raafat AlHakeem, Fahad AlRabiah, Bright Varghese

**Affiliations:** 1 Department of Infection and Immunity, King Faisal Specialist Hospital and Research Centre, Riyadh, Saudi Arabia; 2 Preventive Medicine Directorate, Ministry of Health, Riyadh, Saudi Arabia; 3 Department of Medicine, King Faisal Specialist Hospital and Research Centre, Riyadh, Saudi Arabia; Fundació Institut d’Investigació en Ciències de la Salut Germans Trias i Pujol. Universitat Autònoma de Barcelona. CIBERES, Spain

## Abstract

**Background:**

Bacillus Calmette Guerin (BCG) is the only available vaccine for tuberculosis (TB). Low grade complications in healthy recipients and disseminated vaccine associated complications among immuno-suppressed individuals were noticed globally after administration. Recently a series of clinically suspected BCG associated suppurative and non-suppurative lymphadenitis cases were reported from different regions of Saudi Arabia. However a molecular confirmative analysis was lacking to prove these claims.

**Methodology:**

During 2009–2010, 42 *Mycobacterium bovis BCG* suspected clinical isolates from children diagnosed with suppurative lymphadenitis from different provinces of the country were collected and subjected to 24 loci based MIRU-VNTR typing, spoligotyping and first line anti-TB drugs susceptibility testing.

**Principal Findings:**

Of the total 42 cases, 41 (97.6%) were Saudi nationals and particularly male (64.3%). Majority of the cases were aged below 6 months (83.3%) with a median of age 4 months. All the enrolled subjects showed left axillary mass which suppurated in a median of 4 months after vaccination. Among the study subjects, 1 (2.4%) case was reactive to HIV antigen and 2 (4.8%) case had severe combined immunodeficiency. Genotyping results showed that, 41 (97.6%) isolates were identical to the vaccine strain Danish 1331 and one to Tokyo 172-1. Phylogenetic analysis revealed all the Danish 1331 isolates in a single cluster.

**Conclusion:**

Elevated proportion of suppurative lymphadenitis caused by *M. bovis BCG* reported in the country recently is majorly related to the vaccine strain Danish 1331. However lack of nationwide data on real magnitude of BCG related adverse events warrants population centric, long term future studies.

## Introduction

Bacillus Calmette-Guerin (BCG) is the only available vaccine against *Mycobacterium tuberculosis,* the causative agent of the tuberculosis (TB) since 1921 [1]. It is one of the most common live attenuated vaccines administered around the world and produced from genetically different vaccine strains [2], [3]. In general, local adverse reactions such as administration site abscess and lymphadenitis occur in <1% of healthy recipients [4]. However, disseminated BCG infection and risk of local complications are greatly higher in recipients with congenital immunodeficiency disorders [5]. In addition, high prevalence of human immunodeficiency virus (HIV) makes the situation more complicated [6]. Furthermore, BCG is associated with significant adverse effects, which include the local ulceration at the vaccination site, lymphadenitis, osteomyelitis and variable frequencies of systemic disseminated disease together with regional lymphadenitis causing significant morbidity [6]–[9]. Furthermore, another adverse event namely immune reconstitution inflammatory syndrome has been reported with an unknown etiology among immunocompromised individuals started with antiretroviral therapy [10]. In addition exceptional rare events caused by the BCG vaccine such as sarcoidosis, ocular lesions, erythema nodosum and meningitis also been reported [11].

The use and efficacy of the BCG vaccine is always controversial as the protective efficacy ranged from zero to more than 90% in previous studies [12], [13]. Though the efficacy of the BCG vaccine is controversial, the protection against tuberculous meningitis and miliary tuberculosis in children was generally concurred [14]. The variability in efficacy may stay secondary to genetic variations of different human populations, vaccine strains and the TB exposure background of each patient.

In addition the previous exposure to non-tuberculous mycobacteria has been another important cause associated with variable efficacy induced by BCG immunization [15], [16].

Saudi Arabia is a moderate TB burden country with a mandatory BCG vaccination strategy since 1968. The recent vaccination coverage is 98%. Annually the country reported with an average of 4000 new cases of TB and an incidence rate of 17/100000 populations [17]. Mainly, two different BCG vaccine strains are being used in the country since 2002 namely, Pasteur 1173 P2 and Tokyo 172-1. During 2005, the Danish 1331 strain was introduced for primary use [18]. Interestingly after 2006, two published reports showed a higher rate of BCG related complications in newborns with a highest incidence rate of 3.12 and 10.14 (1.96 before the vaccine change) complications/1000 newborns, respectively and with a predominance of suppurative lymphadenopathy [18], [19]. Both studies proposed that the increasing complication rate might be attributed to the change in vaccine strain since 2005 to the Danish 1331. In addition, Danish 1331 strain is well known for inducing more adverse reactions than any other vaccine strains [20], [21]. However, the available data in previous studies showed the existence of immunocompromised illness also (HIV, severe combined immunodeficiency, Interleukin 12 deficiency) among patients with BCG-associated diseases [18], [19], [22]. There were no laboratory based confirmations on these clinically suspected outbreaks of BCG lymphadenitis in Saudi Arabia. Thus, we designed the current study to use molecular tools to evaluate; the claim of the previous studies that, there is a link between the newly introduced BCG vaccine strain and the outbreak of lymphadenitis among children in the country.

## Materials and Methods

### Study setting

Saudi Arabian national tuberculosis control program established nine central referral laboratories to conduct all sophisticated diagnostic procedures including isolates culture for the suspected TB patients and they collectively serve the 13 provinces of the country. The current study utilized the facilities of these 9 laboratories to enroll the study isolates.

BCG lymphadenitis case in this study was defined as “suppurative lymphadenopathy reported in children less than one year old with a culture positive status”. As this study mainly focused to draw the molecular profiles of the culture isolates to prove the clinically suspected outbreak, only suppurative lymphadenitis cases with *M.bovis BCG* isolate was enrolled. During 2009–2010, mostly based on convenience all the well grown culture isolates from reported suppurative lymphadenitis cases diagnosed from infants were collected. The isolates were transferred to the Mycobacteriology research laboratory of King Faisal specialist hospital and research center in the capital city. Data collection forms were used to accumulate the information from the reference laboratory records on available information on patient’s nationality, age (“age” in this study setting was defined as the actual age of the child, as they all mandatorily received the BCG vaccine in the first 72 hours of their birth), gender, vaccination period, smear positivity and existing predisposing factors including HIV, primary immunodeficiencies or other genetical problems. This study has been reviewed and approved by the research ethics committee of King Faisal specialist hospital and research center. All the data collected for the study was anonymized and no patient identifiers were used throughout data collection and analysis period. As the study utilized data only from the pathology laboratory records and the complete data handling and analysis were carried out anonymously, the need for informed consent was waived by the review committee.

### Laboratory Procedures

Genomic DNA was extracted from all isolates using standard spin column technique (QIAAmp DNA mini kit, Qiagen, Germany). Spoligotyping (Ocimum Biosolutions, Hyderabad, India) and 24 loci based MIRU-VNTR typing (Genoscreen, Cedex, France) were carried out by using the commercially available kits and previously described methods [23], [24]. First line anti-TB drugs (streptomycin, isoniazid, rifampicin and ethambutol) susceptibility testing was carried out by using the automated BACTEC 960 system (Becton Dickinson, CA, USA) with the commercial kit (MGIT SIRE Kit, Becton Dickinson, CA, USA).

Control strains; Four different *M. bovis BCG* strains namely, Danish 1331, Tokyo 172-1, Connaught (all these strains are used for vaccination in Saudi Arabia) and Pasteur P-1173 P2 (VNTR typing control) were used as controls.

### Data analysis

MIRU-VNTR and spoligotyping data were submitted to the international database (www.miru-vntrplus.org) to assign the strain lineages followed by a best match and phylogenetic tree based analysis based on the strategies described previously [25]. A dendogram was used to determine the clustering of strain lineages with MIRU-VNTR typing and spoligotyping signatures based on the unweighted pair group method using average linkages and the categorical coefficient. A strain cluster was defined as two or more isolates sharing completely identical fingerprints. The statistical analysis of the results was carried out by using the software package SPSS V-19.0 (IBM, USA).

## Results

During the study period, 42 isolates of *M. bovis BCG* were collected from 7 of 9 provinces of the country. There were no suppurative cases reported in 2 of the 9 laboratories. However majority of the isolates were enrolled from the Eastern and Central provinces. Demographical data showed, 97.6% of patients as Saudi nationals, dominated by male (64.3%). Majority (83.3%) of the cases were aged between 1–6 months. The median of age was 4 months. Data showed, all the 41 children received 0.05 ml of the Danish 1331 vaccine within 48 hours of their birth. However no data was available on the case which reported with the BCG Tokyo strain.

**Figure 1:**
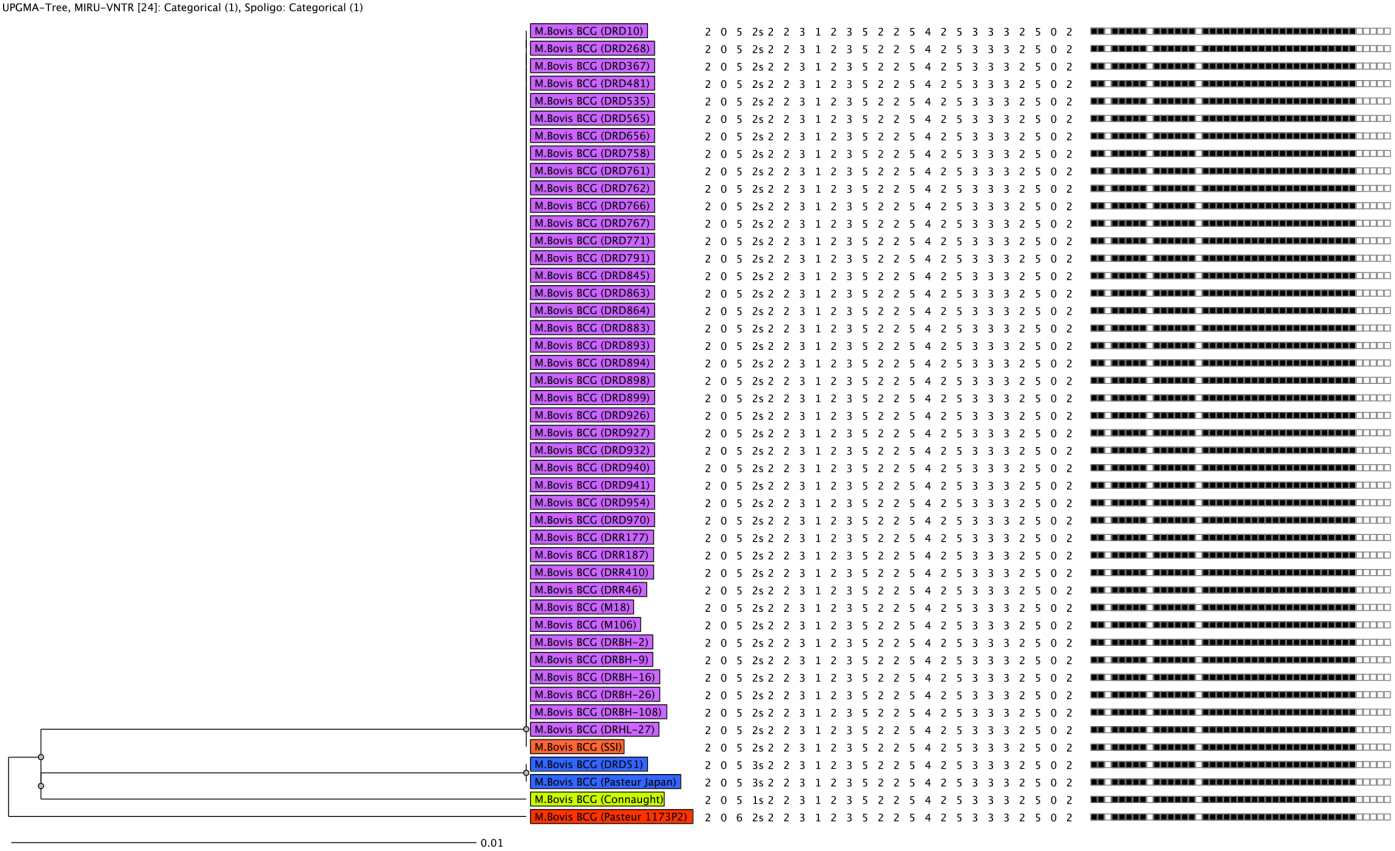
Molecular strain clustering of 42 study isolates and control vaccine strains. The UPGMA tree was built and clusters were identified based on isolates sharing identical MIRU-VNTR types and spoligotypes. Lineages were assigned by best-match analysis followed by tree-based analysis using MIRU-VNTR Plus database. Lineage information followed by isolate identifier number is boxed and colored according to each lineage.

Extra-pulmonary infection was observed in 41 cases (97.6%) whereas one case showed both pulmonary and extra-pulmonary involvement. All the enrolled cases showed isolated left axillary mass which suppurated in a median of 4 months. Susceptibility to first line drugs (SIRE) showed 41 (97.6%) cases were resistant to isoniazid and one case as pan susceptible. HIV reactivity testing results were available only for 11 cases and only 1 case was reactive. Data on genetical predisposing factors were available only for 13 cases. Overall, only two cases of severe combined immunodeficiency (SCID) were noticed (Table 1). Analysis of spoligotyping data showed all the isolates belonged to *M. bovis BCG* assigned with SIT 482 in the database. MIRU-VNTR profiles identified the isolates as *M. bovis.* Further best match analysis with the control strains showed 97.6% (41) of the isolates have similar fingerprints of the vaccine strain BCG Danish 1331. The remaining one belonged to Tokyo 172-1 strain (Figure 1).

**Table 1:** Demographical and Clinical summary of 42 study subjects.

Parametres	Number/percentage
**Nationality**	
Saudi	41 (97.6)
Non Saudi	1 (2.4)
**Age**	
<2 months	9 (21.4)
2–4 months	20 (47.6)
5–6 months	6 (14.3)
>6 months	7 (16.7)
**Gender**	
Male	27 (64.3)
Female	15 (35.7)
**AFB Smear**	
Positive	40 (95.2)
Negative	2 (4.8)
**Predisposing Factors**	
HIV	
Reactive	1 (2.4)
Non-Reactive	10 (23.8)
**PID** a	
SCID^b^	2 (4.8)

a Primary Immunodeficiency.

b Severe combined immunodeficiency.

## Discussion

Saudi Arabia is a moderate TB burden country and follows a mandatory administration of BCG vaccine at birth. Since 2002, the country was using the BCG vaccines prepared from strains Pasteur 1173-P2 and Tokyo 172-1. In 2005, the Danish 1331 vaccine strain was introduced for a nationwide administration. Following this change, several outbreaks of BCG associated lymphadenitis were reported from different institutions in the country [18], [19], [26]. We received a collection of 42 mycobacterial isolates from 7 TB referral laboratories. All isolates were genotyped along with standard vaccine strains (Danish 1331, Connaught and Tokyo 172-1). Data were analyzed together with the available clinical summary. The outcome of the evaluation showed the Danish 1331 strain is associated with the recent rise of mycobacterial lymphadenitis cases among the children.

Demographical data showed a domination of male children. Previous studies also reported high prevalence of lymphadenitis among boys than girls [26]–[28]. The current study showed majorly Saudi nationals. The very little number of non-Saudi cases in the study may due to the small size sampling or increased predominance of such complications among Saudi population. However none of the previous studies considered the nationality of the children for analysis and no such data was available in the country [18], [19], [26]. The drug susceptibility pattern of the isolates showed resistance to isoniazid among the Danish 1331 isolates and pan-susceptibility in Tokyo 172-1 strain. Supportively, the low-level isoniazid resistance of Danish 1331 strain is a known phenomenon [29].

Generally, BCG complications can be induced by the following factors; vaccine strains, viability of bacteria in the final vaccine formulation, overdose, improper administration, vaccination during neonatal period and any type of disturbance to cellular immunity [30], [31]. In addition, an immediate change in vaccine strain before community-based evaluations can also induce the adverse effects. In recent studies, a higher incidence rate of BCG lymphadenitis since 2006 (after introducing Danish 1331 strain) has been observed [18], [19]. The Danish 1331 is known for its high reactogenecity than other vaccine strains [20], [21]. Thus the Danish 1331 strain is reported to incite more adverse reactions than any other strains [32].

The host related factors hold a serious role in the early development of BCG associated complications after vaccination. To date, the primary immunodeficiencies (PID) such as SCID, chronic granulomatous disease (CGD), complete DiGeorge syndrome (cDGS), Mendelian susceptibility to mycobacterial disease (MSMD) and acquired immuno deficiency syndrome (AIDS) are well characterized as predisposing factors for BCG associated complications particularly in disseminated infection [33]–[38]. Saudi Arabian population is highly unique with the highest rate of consanguinity (58%) in the world and significant association of genetic disorders [39], [40]. Interestingly the prevalence of most of the PID’s predisposing BCG infection within the Saudi Arabian population is very much higher (CGD- 5.2 cases/100000 live births, SCID −19 cases/100000 live births) than any other population in the world [41], [42].

Surprisingly, SCID incidence rate in Saudi Arabia is the highest in the world and 20 times more than the incidence rate of European countries [41]. The current study also reported with two cases of SCID among the study subjects. Another previous study clearly established the role of SCID in BCG related complications in Saudi Arabian children, which even include disseminated form of BCGitis [22]. The increasing number of MSMD cases in the country can predispose as a prominent factor for the BCG complications as it recently reported with a gradual increase around the world with huge presence in Saudi Arabia [43], [44]. Furthermore, previous BCG lymphadenitis outbreak reports also accounted with cases of Interleukin 12 deficiencies [19]. There was only one case of HIV reactivity reported in this study. However, the congenital transmission of 4% HIV cases among Saudi population even in the current moderate rate of prevalence (<4cases/100000 populations) is a serious concern [45]. The undisputed role of HIV/AIDS positivity in inducing the BCG infections in the country is highly considerable as it already established in other settings [6].

This study has certain limitations; due to lack of complete access to the patient’s records (limited data made only available from pathology laboratory records), data collection on clinical history was merely complete. Enrollment of culture positive cases alone limited an estimation of a nationwide prevalence of BCG lymphadenitis. Due to the exclusion of certain defined cases without a positive culture an unquantifiable selection bias was expected. This is mainly due to the limitations on data collection from all the case notified hospitals. Unavailability of clinical samples restricts exploring the genetical and immunological disorders in detail and which was beyond the scope of the study.

As previous reports showed, recent change in vaccine strain without any proper evaluation of admissibility led to the higher incidence of BCG associated lymphadenitis [18], [19]. There were no available data on the real potential of BCG vaccination among Saudi population, while there are increasing incidences of immunodeficiency disorders among Saudi Arabian children. Indeed, an immediate evaluation on, the need and efficacy of BCG in the country is proposed. The mandatory BCG vaccination policy was made when the annual incidence rate of TB was 243cases/100000 populations and that gradually decreased to 22 cases/100000 populations by now. In addition it is highly advisable to reconsider the routine BCG vaccination time which is contraindicated in immunosuppressed/immune-deficient children. Furthermore, the host related risk factors needed to be explored in detail. Thus a longitudinal long-term study to explore the effects of genetic and immunological factors on mycobacterial disease activation and transmission is an absolute necessity in the country.
